# Utilization of the Disease Severity Index (DSI) from the HepQuant DuO Test Enhances Clinical Decision Making in Compensated Advanced Chronic Liver Disease

**DOI:** 10.3390/jcm15020501

**Published:** 2026-01-08

**Authors:** Kerry Whitaker, Joanne C. Imperial, Michael P. McRae, Gregory T. Everson

**Affiliations:** 1 HepQuant, LLC, Denver, CO 80237, USA; 2 Custom DX Solutions LLC, Houston, TX 77005, USA

**Keywords:** liver function, clinical utility, disease severity index, portal-systemic shunting, portal hypertension, varices, compensated advanced chronic liver disease

## Abstract

**Background/Objectives:** Compensated advanced chronic liver disease (cACLD) affects millions and carries risk for portal hypertension, large varices, and clinical decompensation. The HepQuant DuO^®^ test provides a blood-based assessment of liver function and physiology, generating a disease severity index (DSI) validated for risk stratification. A retrospective, real-world, observational analysis was conducted to evaluate the utility of HepQuant DuO’s DSI cutpoint (18.3) in guiding endoscopy and clinical management decisions for patients with cACLD in the United States. **Methods:** De-identified data from 87 cases with cACLD were extracted from physician-provided Statements of Medical Necessity documenting the reasons for the HepQuant DuO test. The primary endpoint was concordance of endoscopy decisions with DSI ≤ 18.3 (avoid) or >18.3 (proceed). The secondary endpoint was concordance of clinical management intensity with the same cutpoint. **Results:** Among the 55 cases analyzed for endoscopy decisions, overall concordance with DSI 18.3 was 93% (*p* < 0.001 by Fisher’s exact test): 96% of cases with DSI ≤ 18.3 avoided endoscopy, and 90% with DSI > 18.3 underwent endoscopy. For the 45 cases assessing management intensity, overall concordance was 89% (*p* < 0.001): 90% of cases with DSI ≤ 18.3 had reduced follow-up, and 89% with DSI > 18.3 had intensified management. The cohort exhibited broad functional heterogeneity not captured by standard labs or elastography. **Conclusions:** HepQuant DuO’s DSI cutpoint 18.3 demonstrated strong concordance with real-world clinical decisions, supporting its utility for risk stratification, optimizing endoscopy use, and tailoring management in cACLD.

## 1. Introduction

Compensated advanced chronic liver disease (cACLD) encompasses patients with advanced fibrosis and patients with compensated cirrhosis, affects millions of Americans, and is responsible for more than 50,000 deaths occurring each year through 2023 [1,2]. Patients with cACLD are at risk for large varices and variceal hemorrhage, clinical decompensation, liver-related death, and hepatocellular carcinoma [3]. Identifying patients with cACLD who are at risk for these clinical outcomes is a priority of clinical management.

Elastography with platelet count has been the main noninvasive strategy for defining clinical risk [2,4,5,6,7]. The accuracy of this noninvasive approach is compromised in patients who are overweight, obese, diabetic, elderly, and have metabolic dysfunction-associated steatotic liver disease (MASLD) or steatohepatitis (MASH) [8,9,10,11,12]. Additional noninvasive radiologic and blood-based methods are currently under investigation [13].

The HepQuant DuO test is a noninvasive, blood-based test that assesses global liver health by quantifying liver function and physiology [14,15]. The test generates a disease severity index (DSI) for assessment of risk for portal hypertension and large esophageal varices (LEV) to aid in the upper endoscopy (EGD) decision [16], provides a definition of disease severity to aid in clinical management, and enables serial testing to monitor changes in liver health over time, either improvement or worsening.

A DSI cutpoint 18.3 was defined in a U.S. multi-center trial in advanced chronic hepatitis C (HALT-C) [17] and validated in a second U.S. multi-center trial where the majority of cases (52%) had MASLD/MASH (SHUNT-V) [16]. In addition, the latter validation study included all common etiologies of cACLD, and a high percentage of the study subjects were overweight, obese, elderly, and had diabetes. In several studies, DSI has shown favorable diagnostic performance compared to other noninvasive tests [16,18].

Using real-world data, the analysis evaluated the impact of DSI 18.3 in the EGD decision and in modifying decision making in patients with cACLD.

## 2. Methods

In this retrospective analysis, de-identified information was extracted from Statement of Medical Necessity (SMN) letters received from ordering physicians from 23 practices (academic and community) in the United States to support the medical necessity of the HepQuant DuO test. The SMN letters addressed two key decision-making parameters: decision to avoid or proceed with EGD to evaluate large esophageal varices, and decision to reduce or enhance the intensity of clinical management and follow-up, defined as frequency of clinic visits and laboratory assessments. All personal identifiers were removed prior to analysis. The physician-signed HepQuant test requisition form authorized use of de-identified information by HepQuant for research purposes.

The HepQuant DuO test was ordered as part of routine clinical practice. Blood collection was performed after a period of fasting greater than 5 h. Nonradioactive d4-cholate was taken orally in aqueous solution followed by drawing of peripheral venous blood samples at 20 and 60 min. Samples were allowed to clot, centrifuged, and the serum was transferred to cryovials for shipment to HepQuant’s laboratory for analysis. The HepQuant laboratory is the sole source for the cholate assay and is certified under the Clinical Laboratory Improvement Amendments (CLIA).

**Figure 1:**
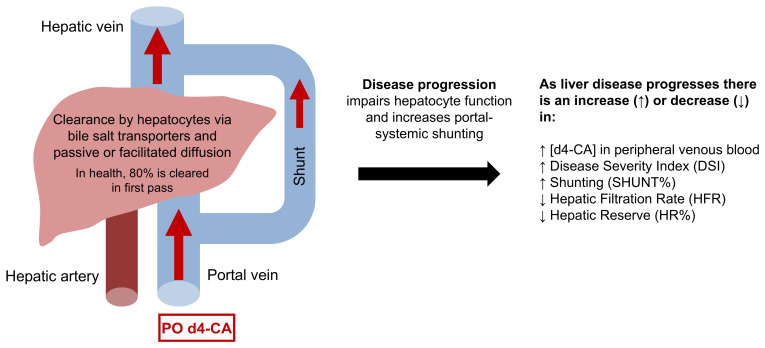
Overview of the HepQuant DuO^®^ test of liver function and physiology.

HepQuant DuO assesses hepatocyte function (uptake of cholate), liver blood flow (cholate clearance is flow-dependent), and portal-systemic shunting (Figure 1). The primary output of the HepQuant DuO test is DSI, a proprietary score indexing a patient’s cholate clearances against maximum clearances of healthy controls. Other test parameters included the following:

- Hepatic filtration rates (HFR) are cholate clearances adjusted for body weight. Portal HFR is the clearance of orally administered d4-cholate, and systemic HFR is the derived systemic clearance of cholate [14].
- SHUNT% is the portal-systemic shunt fraction, which is defined as the ratio of the systemic and portal clearances.
- Hepatic reserve (HR%) indexes a patient’s cholate clearances against the lower limit of clearances of healthy controls of lean body mass.

The results were retrieved from the test reports and verified from official stored documents within the HepQuant Laboratory Information Management System (LIMS). Collected materials included the HepQuant DuO test reports, SMN letter responses, and other clinical information such as the ordering physician’s name and accompanying clinic notes from the test order or received with the SMN letters.

Subject characteristics, laboratory values, and HepQuant DuO test results were reported for all cases that had completed a HepQuant DuO test. In the same cohort, the spectrum of function (DSI) and shunting (SHUNT%) was plotted, and pairwise correlations between laboratory values, noninvasive tests, and DSI were evaluated.

Decisions were analyzed by DSI 18.3, a validated cutpoint for risk of portal hypertension and large esophageal varices [16]. The primary endpoint was the concordance of the decision to avoid or proceed with EGD to check for varices and other lesions of portal hypertension based upon DSI 18.3. Concordance was defined as avoidance of EGD when DSI ≤ 18.3 and proceeding to EGD when DSI > 18.3. A secondary endpoint was the concordance of changes in clinical management, defined by either increased or decreased intensity of clinical follow-up, based upon DSI 18.3. Here, concordance was defined as decreased intensity of clinical management when DSI ≤ 18.3 and increased intensity of clinical management with DSI > 18.3. Results were displayed in 2 × 2 tables, and the significance of the concordance of clinical endpoints with the DSI cutpoint was evaluated by Fisher’s exact test.

## 3. Results

### 3.1. Statements of Medical Necessity

SMN letters (n = 92) were received from ordering physicians from 16 June to 13 October 2025. Three of the SMN letters were duplicates and were excluded, resulting in a total of 89 cases with completed HepQuant DuO tests. A total of 2 cases were excluded for incomplete information, resulting in a total of 87 cases with evaluable data. The intended uses for HepQuant DuO in these 87 cases were the EGD decision in 34, clinical management in 14, both EGD decision and clinical management in 31, and others in 8.

### 3.2. Demographics and Selected Characteristics

Selected characteristics (Table 1) included the following: male/female ratio of 36:53, age range 32 to 84, average age 65 ± 12 years, 66% overweight, 34% obese, average BMI 27.9 ± 5.6 kg/m^2^, and 31.5% with MASLD/MASH. Other less common etiologies included cryptogenic cirrhosis, alcohol-associated liver disease (ALD), autoimmune hepatitis, Met-ALD, and hepatitis C. All cases met criteria for cACLD.

### 3.3. Baseline Functional and Physiological Heterogeneity of the Population

**Figure 2:**
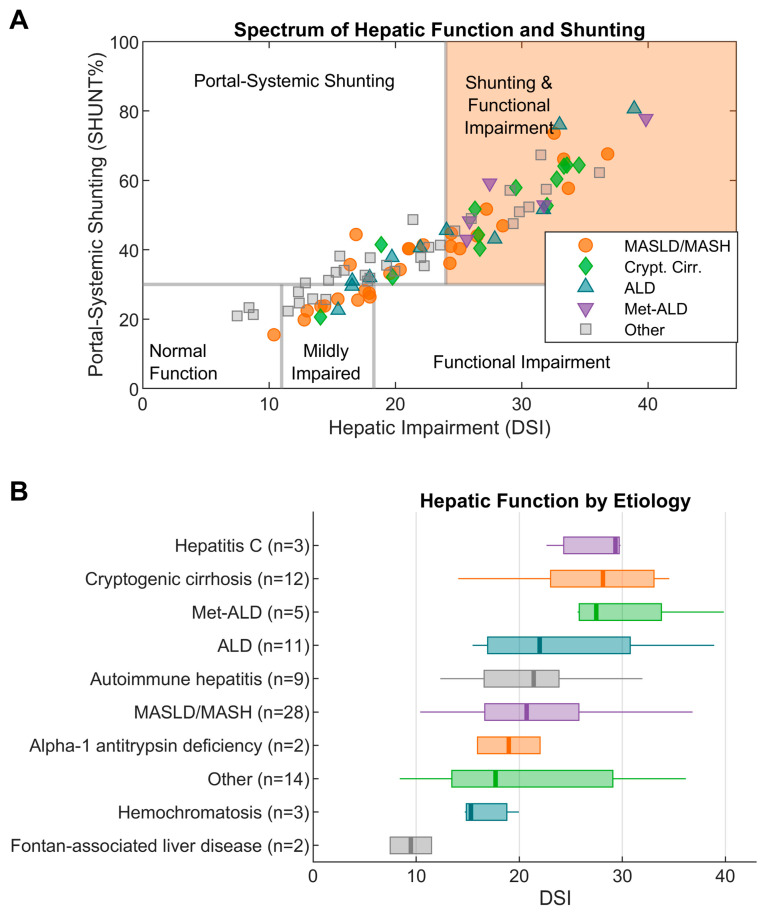
Hepatic function and shunting across all cases with completed HepQuant DuO tests (n = 89) by liver disease etiology (**A**); hepatic function by etiology sorted by median DSI (**B**).

Figure 2 is a plot of DSI versus SHUNT% for the 89 cases with completed HepQuant DuO tests. Categories of disease severity from low impairment to severe impairment are based on cutoffs established in published studies of the long-term follow-up of patients with cACLD who had baseline testing [19]. As seen in other published studies of HepQuant DuO [16,20], DSI and SHUNT% uncover a broad spectrum of hepatic impairment in a cACLD population with essentially similar and normal standard liver chemistries. Although standard test results were not available for all patients, there were 43 cases where standard lab tests had been drawn within 6 months of the HepQuant DuO test (Supplemental Table S1). These standard test results indicated that this cohort was well-compensated: bilirubin 1.3 ± 0.8 mg/dL, albumin 4.0 ± 0.6 g/dL, INR 1.2 ± 0.1, platelet count 126 ± 66 per nL, and creatinine 0.9 ± 0.3 mg/dL. In contrast to these standard test results, DSI and SHUNT% uncovered a wide range of hepatic impairment, ranging from mild to severe.

### 3.4. Correlation of DSI, SHUNT%, and HR% with Bilirubin and Platelet Count

Standard laboratory tests were largely within normal limits in this well-compensated cACLD cohort (Supplemental Table S1). Nevertheless, bilirubin and platelet count showed moderate but statistically significant correlations with HepQuant DuO parameters (Supplemental Figures S1–S3). Bilirubin correlated positively with DSI (*r* = 0.53, *p* < 0.01) and SHUNT% (*r* = 0.62, *p* < 0.01), and inversely with HR% (*r* = −0.53, *p* < 0.01). Platelet count correlated inversely with DSI (*r* = −0.59, *p* < 0.001), SHUNT% (*r* = −0.52, *p* < 0.001), and positively with HR% (*r* = 0.56, *p* < 0.001). In contrast, vibration-controlled transient elastography (VCTE) data were available in only 17 cases, and neither liver stiffness (kPa) nor controlled attenuation parameter (CAP) correlated significantly with standard laboratory values, DSI, SHUNT%, or HR% (Supplemental Figure S4). These findings underscore the limited ability of conventional blood tests and elastography to capture the broad spectrum of physiological impairment detected by the HepQuant DuO test in compensated patients.

### 3.5. DSI 18.3 and the Endoscopy Decision

In 65 cases, the SMN letter indicated that the HepQuant DuO test result was used for deciding to avoid or proceed with EGD. Of these, 10 had EGD prior to the availability of the DSI result, leaving 55 cases for analysis of the impact of DSI on the EGD decision.

The 2 × 2 table in Table 2 displays the results for performance of EGD stratified by the DSI cutpoint 18.3. A total of 24 of the 25 cases with DSI ≤ 18.3 avoided EGD, indicating 96% concordance of the DSI result with the subsequent clinical decision to avoid EGD. Furthermore, 27 of the 30 cases with DSI > 18.3 proceeded to EGD, indicating 90% concordance of the DSI result with the subsequent clinical decision to proceed to EGD. The overall concordance of the DSI result, for both avoiding and proceeding with EGD, was 93% (*p* < 0.001 by Fisher’s exact test).

EGD was performed in 28 cases, 27 of which had DSI > 18.3 (96%). EGD was avoided in 27 cases, 24 of which had DSI ≤ 18.3 (89%). Fifty-five percent (30/55) of cases had DSI > 18.3.

One case with DSI ≤ 18.3 underwent EGD, and three cases with DSI > 18.3 did not undergo EGD. In these instances, EGD decisions preceded the availability of the DSI results, or a recent EGD had already been completed before DSI testing.

### 3.6. DSI 18.3 and the Intensity of Clinical Management

In 45 cases, the SMN letter indicated that the HepQuant DuO test result was used for reducing or enhancing clinical management and follow-up. Table 3 displays the 2 × 2 results for a DSI cutoff of 18.3 used to determine whether to increase or decrease clinical follow-up intensity. The data are analyzed for the use of DSI 18.3 in the decisions to increase or decrease the intensity of clinical management. A total of 9 out of 10 cases with DSI ≤ 18.3 received less intensive clinical management and follow-up, representing 90% concordance of the DSI result with the subsequent clinical decision to reduce follow-up intensity. A total of 31 of 35 cases with DSI > 18.3 had more intense clinical follow-up, representing 89% concordance of the DSI result with the subsequent clinical decision to increase intensity of clinical follow-up. The overall concordance of the DSI result for both increasing and decreasing intensity of clinical follow-up was 89% (*p* < 0.001 by Fisher’s exact test).

Clinical management was intensified in 32 cases, 31 of which had DSI > 18.3 (97%). Intensity of follow-up was decreased in 13 cases, 9 of which had DSI ≤ 18.3 (69%). Seventy-seven percent (35/45) had DSI > 18.3.

One case that had DSI ≤ 18.3 had increased intensity of clinical follow-up, and four cases that had DSI > 18.3 did not have increased intensity of clinical follow-up, since DSI results had not returned in time for altering the management decisions.

The proportion of cases with DSI > 18.3 was greater in the care-management group (77%) compared to the EGD-decision group (55%). The higher proportion with DSI > 18.3 confirmed appropriate selection of cases needing more intense clinical follow-up.

## 4. Discussion

This retrospective analysis is the first to evaluate the utility of the HepQuant DuO test for clinical decision making in patients with cACLD. The primary predictive variable is the disease severity index, DSI from the HepQuant DuO test, with a cutpoint of 18.3. The cutpoint DSI 18.3 was defined from an analysis of U.S. patients with cACLD due to chronic hepatitis C (HALT-C Trial, Quantitative Liver Function Test [QLFT] ancillary study) [17] and validated for prediction of risk for clinically significant portal hypertension and LEV (SHUNT-V) with leading etiology of MASH cACLD [16].

The diagnostic performance of the DSI cutpoint of 18.3 for ruling out LEV indicated a sensitivity of 98% (95% CI: 89–100%), negative predictive value of 99% (95% CI: 97–100%), and negative likelihood ratio of 0.04 (95% CI: 0.01–0.31) [16]. Patients with a DSI ≤ 18.3 would be at very low risk for LEV, and the physician could use this information to support a decision to forego or defer EGD. If applied in practice, up to 41.3% of unnecessary EGDs could be prevented [16]. This is the basis for the use of DSI 18.3 in clinical practice to inform the decision to avoid EGD.

The real-world data depicted in the current analysis demonstrated that physicians used DSI ≤ 18.3 to aid in the decision to forgo EGD and to reduce intensity of clinical management (i.e., decrease frequency of follow-up visits or laboratory measurements). The outcomes from decisions related to DSI ≤ 18.3 could include avoidance of the inconvenience, discomfort, and cost of unnecessary clinic visits, laboratory tests, and other studies. Conversely, physicians used the DSI > 18.3 to aid in the decision to proceed with EGD and to increase the intensity of clinical care (i.e., increase frequency of follow-up visits or laboratory measurements). The outcomes from decisions related to DSI > 18.3 could include avoidance of unanticipated variceal hemorrhage and lowering risk for decompensation, hospitalization, and mortality.

**Figure 3:**
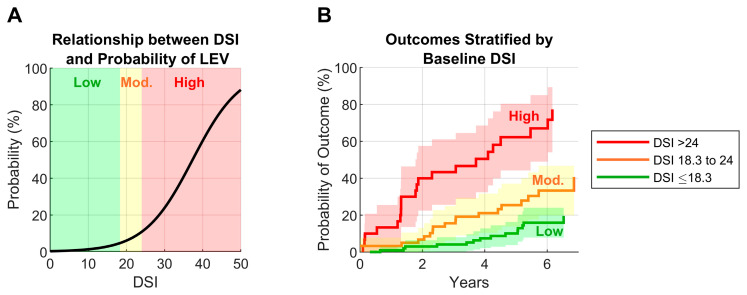
Relationship between the disease severity index (DSI) and large esophageal varices (LEV) and clinical outcome. DSI risk categories are defined as low risk (DSI ≤ 18.3), moderate risk (DSI 18.3 to 24), and high risk (DSI > 24). (**A**) shows the logistic regression function linking DSI to the probability of finding LEV at endoscopy in HALT-C and SHUNT-V studies (n = 455) [16]. (**B**) shows Kaplan–Meier curves expressing the probability of clinical outcome over time stratified by DSI in 195 subjects with ongoing chronic hepatitis C who had no or small varices [19].

A broad range of functional heterogeneity was observed within cACLD (Figure 2), ranging from normal function to severe functional impairment and shunting. This pattern is consistent with observations in over 700 patients with HepQuant DuO in prior clinical studies, highlighting the ongoing need for function testing in the management of cACLD. As shown in Figure 3, the probabilities of LEV [16] and clinical outcome [19] increase exponentially above the DSI cutpoint 18.3. The physician could use the probability of LEV based on DSI (Figure 3A) as supportive information in the decision to avoid or proceed with EGD. Likewise, the physician could individualize their patient’s follow-up plan according to the risk for clinical outcome over time based on DSI (Figure 3B). Incorporating quantitative liver function testing into routine care may enable more precise, risk-based decisions for endoscopy and longitudinal management in patients with cACLD.

Non-selective β-blockers (NSBBs) are increasingly used in managing cACLD, not only for reducing variceal bleeding risk but also for preventing first hepatic decompensation in patients with clinically significant portal hypertension (CSPH). The Baveno VII consensus recommends noninvasive assessment of CSPH status by liver stiffness measurements (LSM) by VCTE and platelet count [4]. Global adoption of this empiric approach remains variable. In Europe, despite limited data on its implementation in real-world clinical practice, the Baveno VII consensus recommendations are officially endorsed by the European Association for the Study of the Liver (EASL) [21]. In Asia, adoption of these recommendations is mixed, with some clinicians favoring endoscopic confirmation due to variability in VCTE access and concerns of the reliability of noninvasive tests as surrogates of CSPH [22].

In the United States, the American Association for the Study of Liver Diseases (AASLD) recommendation generally aligns with Baveno VII in that noninvasive testing by platelet count and LSM may be used to identify CSPH. If CSPH is present, non-selective beta-blocker (NSBB) therapy should be initiated to reduce the risk of decompensation [2]. However, in this analysis representing real-world clinical decision making in the United States in 2025, only 17 of 89 (19%) cases had LSM reported. This experience was comparable to the prospective, multi-center, U.S. pivotal study conducted between 2019 and 2021 in patients with Child–Pugh A cirrhosis scheduled for EGD as part of standard of care (SHUNT-V) [16]. This analysis found that only 78 of 238 (33%) had LSM results reported within one year of enrollment [16]. These findings suggest that the Baveno VII recommendations may not be used routinely by U.S. gastroenterologists or hepatologists in the decision-making process, whether for EGD or empiric NSBB therapy.

One practical limitation contributing to the slow and fragmented adoption of Baveno VII in the U.S. is the reduced accuracy and feasibility of LSM in patients with obesity. Patients with obesity represent a large and growing proportion of cACLD, and MASLD/MASH is the predominant liver disease etiology. In contrast to LSM, the HepQuant DuO test is unaffected by obesity, with no significant differences in the diagnostic accuracy for LEV across BMI categories.

Empiric NSBB therapy carries risks and potential harm without benefit in patients misclassified as having CSPH—concerns that are amplified when LSM accuracy is limited, especially in obesity. Additionally, measuring the efficacy of NSBB therapy is challenging, requiring highly invasive procedures like hepatic venous portal pressure gradient (HVPG). As a result, clinicians may hesitate to initiate NSBB therapy when LSM-based CSPH status is uncertain.

Contrary to LSM, the HepQuant DuO test is agnostic to liver disease etiology and uncovers risk for LEV and clinical outcome not revealed by standard assessments [18]. For example, in a Veterans Administration cirrhotic population, only 33% of patients meeting standard of care guidelines for EGD to check for LEV actually underwent EGD, and there was a high rate of clinical outcome and mortality within a median follow-up of 6.1 years [23]. This experience highlights the need for improvement in risk assessment as provided by the DSI.

This analysis has several limitations. First, the sample size was relatively small, and not every patient had complete information regarding EGD, which limited the evaluation of decision concordance in some cases. Second, standard laboratory results and LSM were not consistently available, limiting comparisons between HepQuant DuO parameters and other noninvasive markers. Third, the study was confined to centers in the United States, which may reduce generalizability to other healthcare systems and practice patterns outside of the U.S. Additionally, the cohort was derived from physicians who elected to order the HepQuant DuO test, which may represent selection bias based on a subset of clinicians more inclined to agree with the results from advanced diagnostic tools. These factors should be considered when interpreting the findings and planning future prospective studies. Finally, for full clinical utility, a prospective study of the impact of HepQuant DuO results on decision making and linking these decisions to changes in performance of EGD and clinical management is needed.

In conclusion, this analysis supports the use of the HepQuant DuO test for clinical decision making in patients with cACLD. Our findings demonstrated that physicians, particularly specialists caring for patients with cACLD, used the DSI cutpoint 18.3 to guide decisions on whether to avoid or proceed with EGD and to determine the appropriate intensity of clinical management and follow-up.

## Figures and Tables

**Table 1:** Selected characteristics, liver disease etiologies, and HepQuant DuO^®^ test parameters across all cases (n = 89).

	Mean ± SD or n (%)	Min.	Max.
Characteristics			
Age (years)	64.9 ± 11.6	32	84
Male	36 (40.4)	-	-
Body mass index (kg/m^2^)	27.9 ± 5.6	14.8	43.4
Overweight	59 (66.2)	-	-
Obese	30 (33.7)	-	-
Liver disease etiologies			
MASLD/MASH	28 (31.5)	-	-
Cryptogenic cirrhosis	12 (13.5)	-	-
Alcohol-associated liver disease	11 (12.4)	-	-
Autoimmune hepatitis	9 (10.1)	-	-
Met-ALD	5 (5.6)	-	-
Hepatitis C	3 (3.4)	-	-
Hemochromatosis	3 (3.4)	-	-
Alpha-1 antitrypsin deficiency	2 (2.2)	-	-
Fontan-associated liver disease	2 (2.2)	-	-
Nonregenerative hyperplasia	1 (1.1)	-	-
Primary sclerosing cholangitis	1 (1.1)	-	-
Other	12 (13.5)	-	-
HepQuant DuO^®^ test parameters			
DSI	22.5 ± 7.7	7.5	39.8
SHUNT% (%)	41.6 ± 15.0	15.5	80.6
Hepatic reserve (%)	70.9 ± 19.4	26.4	100
Cases with DSI > 24 and SHUNT% > 30%	39 (43.8)	-	-

Abbreviations: DSI, disease severity index; Min., minimum; Max., maximum; SD, standard deviation; SHUNT%, portal-systemic shunting.

**Table 2:** 2 × 2 table showing the impact of DSI on the decision to perform EGD.

	EGD	No EGD	Totals
DSI > 18.3	27	3	30
DSI ≤ 18.3	1	24	25
Totals	28	27	55

**Table 3:** 2 × 2 table showing the impact of DSI on the clinical decision to increase or decrease follow-up intensity.

	Increased	Decreased	Totals
DSI > 18.3	31	4	35
DSI ≤ 18.3	1	9	10
Totals	32	13	45

## Data Availability

Data from individual cases will not be shared.

## Supplementary Materials

The following supporting information can be downloaded at https://www.mdpi.com/article/10.3390/jcm15020501/s1, Table S1. Laboratory values collected within 6 months of the HepQuant DuO test and noninvasive tests; Figure S1. Pairwise correlation coefficients for laboratory values, noninvasive tests, and disease severity index (DSI); Figure S2. Relationship of DSI to platelet count. Platelet count declines as DSI increases, but the strength of the relationship is weak. Most of the patients with DSI > 24 had thrombocytopenia; but thrombocytopenia was also common in the cases with DSI < 24; Figure S3. Relationship of DSI to bilirubin. Bilirubin increases as DSI increases, but the strength of the relationship is weak. Most of the increases in bilirubin occurred in patients with DSI > 24, consistent with bilirubin being a marker primarily for late-stage disease; Figure S4. Relationship of DSI to liver stiffness measurement by FibroScan. There was no correlation of kPa from FibroScan with DSI. Of the 5 with high risk (kPa > 25) for clinically significant portal hypertension (CSPH) and varices, 2 had either low or intermediate risk DSI. The one with kPa between 15 to 25, indicating high likelihood of cACLD, had intermediate risk DSI. Of the 9 with kPa < 15 and low risk for CSPH and avoidance of EGD, 6 had either intermediate or high risk DSI.

## Abbreviations

cACLD—Compensated Advanced Chronic Liver Disease; MASLD—Metabolic Dysfunction–Associated Steatotic Liver Disease; MASH—Metabolic Dysfunction–Associated Steatohepatitis; DSI—Disease Severity Index; LEV—Large Esophageal Varices; EGD—Esophagogastroduodenoscopy (Endoscopy); SMN—Statement of Medical Necessity; CLIA—Clinical Laboratory Improvement Amendments; HFR—Hepatic Filtration Rate; SHUNT%—Portal-Systemic Shunt Fraction; HR%—Hepatic Reserve; HALT-C—Hepatitis C Antiviral Long-Term Treatment Against Cirrhosis Trial; SHUNT-V—Multi-Center U.S. Cohort Validation Study; Met-ALD—Metabolic Alcohol-Associated Liver Disease; ALT—Alanine Transaminase; AST—Aspartate Aminotransferase; CAP—Controlled Attenuation Parameter; NSBB—Non-Selective β-Blockers; CSPH—Clinically Significant Portal Hypertension; LSM—Liver Stiffness Measurement; EASL—European Association for the Study of the Liver; AASLD—American Association for the Study of Liver Diseases; HVPG—Hepatic Venous Portal Pressure Gradient.
