# Supplementary material for: Utilization of the Disease Severity Index (DSI) from the HepQuant DuO Test Enhances Clinical Decision Making in Compensated Advanced Chronic Liver Disease
Source: J Clin Med. 2026 Jan 8;15(2):501. doi: 10.3390/jcm15020501 (PMC12841770; doi:10.3390/jcm15020501)
Supplement: Supplementary file 1 [file jcm-15-00501-s001.zip › jcm-4068343-supplementary.pdf]

Supplemental Materials

Table S1. Laboratory values collected within 6 months of the HepQuant DuO test and noninvasive tests

	n	Mean ± SD or n (%)	Min.	Max.
**Laboratory values**				
Albumin (g/dL)	31	4.0 ± 0.6	2.6	5.1
Alkaline phosphatase (U/L)	29	140.8 ± 82.6	57	348
ALT (U/L)	40	38.4 ± 24.2	10	103
AST (U/L)	41	47.0 ± 23.9	18	155
Total bilirubin (mg/dL)	31	1.3 ± 0.8	0.2	3.6
Creatinine (mg/dL)	32	0.9 ± 0.3	0.4	1.5
Hematocrit (%)	26	39.5 ± 5.2	27.6	47.9
Hemoglobin (g/dL)	27	12.9 ± 2.0	9	16.1
International normalized ratio	18	1.2 ± 0.1	1	1.4
Platelet count (nL^{-1})	38	126 ± 66	32	300
Prothrombin time (sec)	14	13.4 ± 1.9	11.3	18
Sodium (mEq/L)	28	138.4 ± 2.6	130	144
White blood cell count ($\times 10^3$)	28	5.2 ± 2.1	1.9	12.4
**Noninvasive tests**				
Liver stiffness (kPa)	17	21.4 ± 15.6	6.1	62
CAP score (dB/m)	11	253.2 ± 95.3	21	344

[†] One case with acute alcohol-associated hepatitis was excluded.

Abbreviations: ALT, alanine transaminase; AST, aspartate aminotransferase; CAP, controlled attenuation parameter; Min., minimum; Max., maximum; SD, standard deviation.

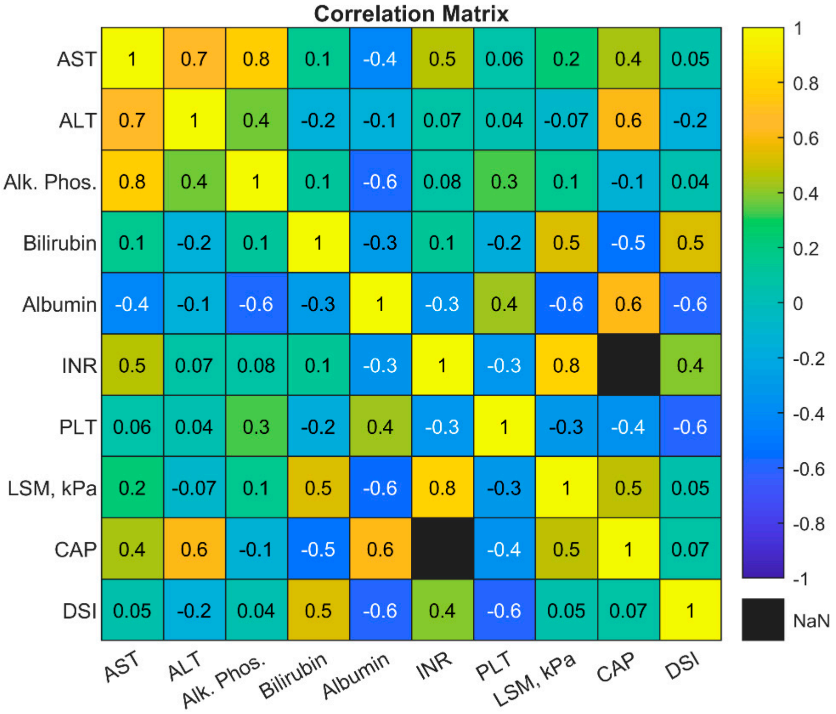

Figure S1. Pairwise correlation coefficients for laboratory values, noninvasive tests, and disease severity index (DSI).

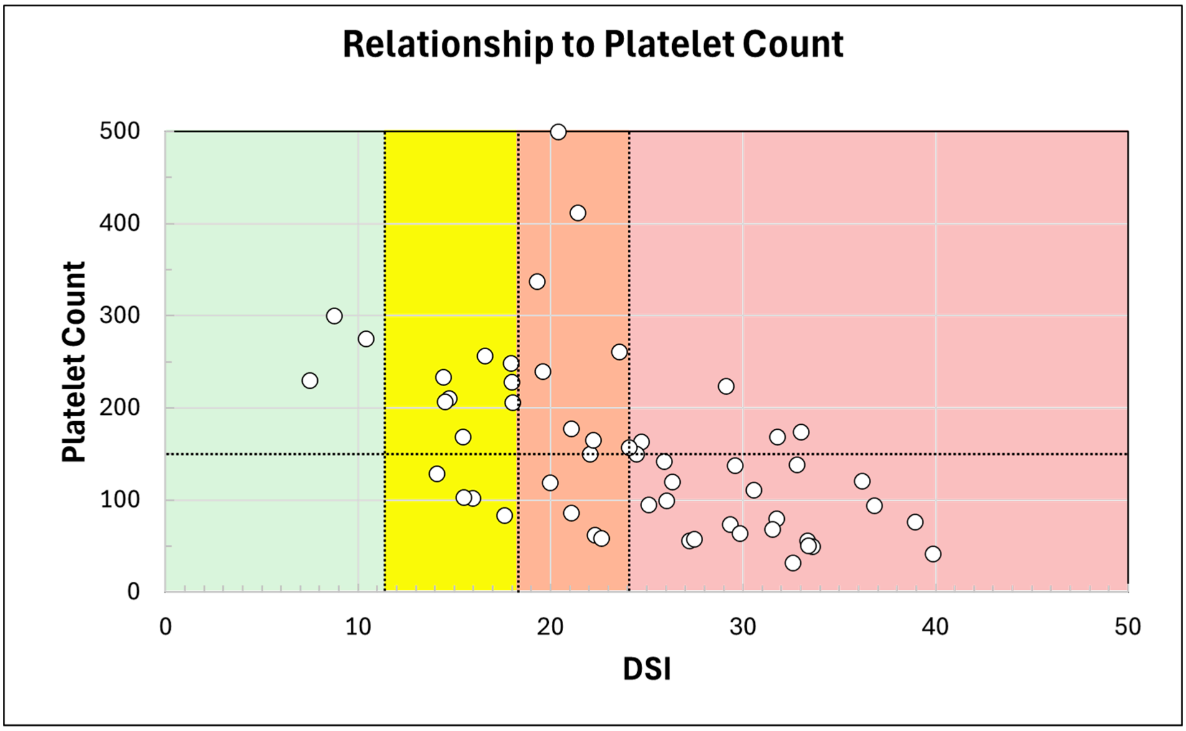

Figure S2. Relationship of DSI to platelet count. Platelet count declines as DSI increases, but the strength of the relationship is weak. Most of the patients with DSI >24 had thrombocytopenia; but thrombocytopenia was also common in the cases with DSI <24.

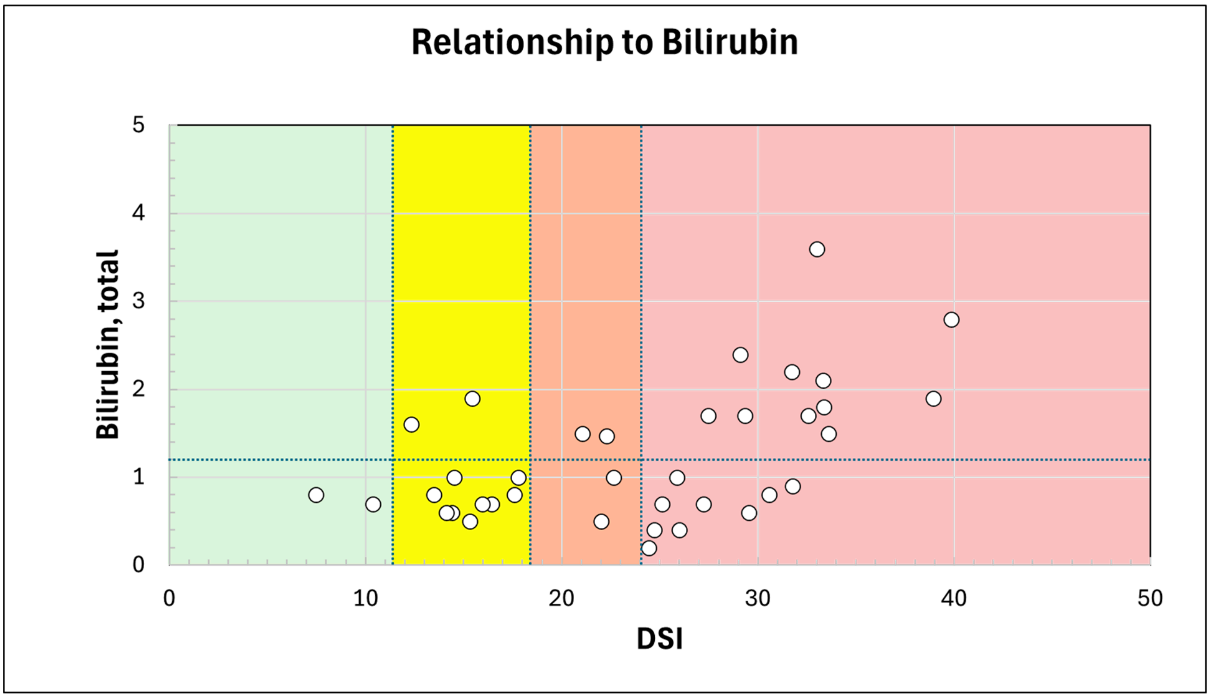

Figure S3. Relationship of DSI to bilirubin. Bilirubin increases as DSI increases, but the strength of the relationship is weak. Most of the increases in bilirubin occurred in patients with DSI >24, consistent with bilirubin being a marker primarily for late-stage disease.

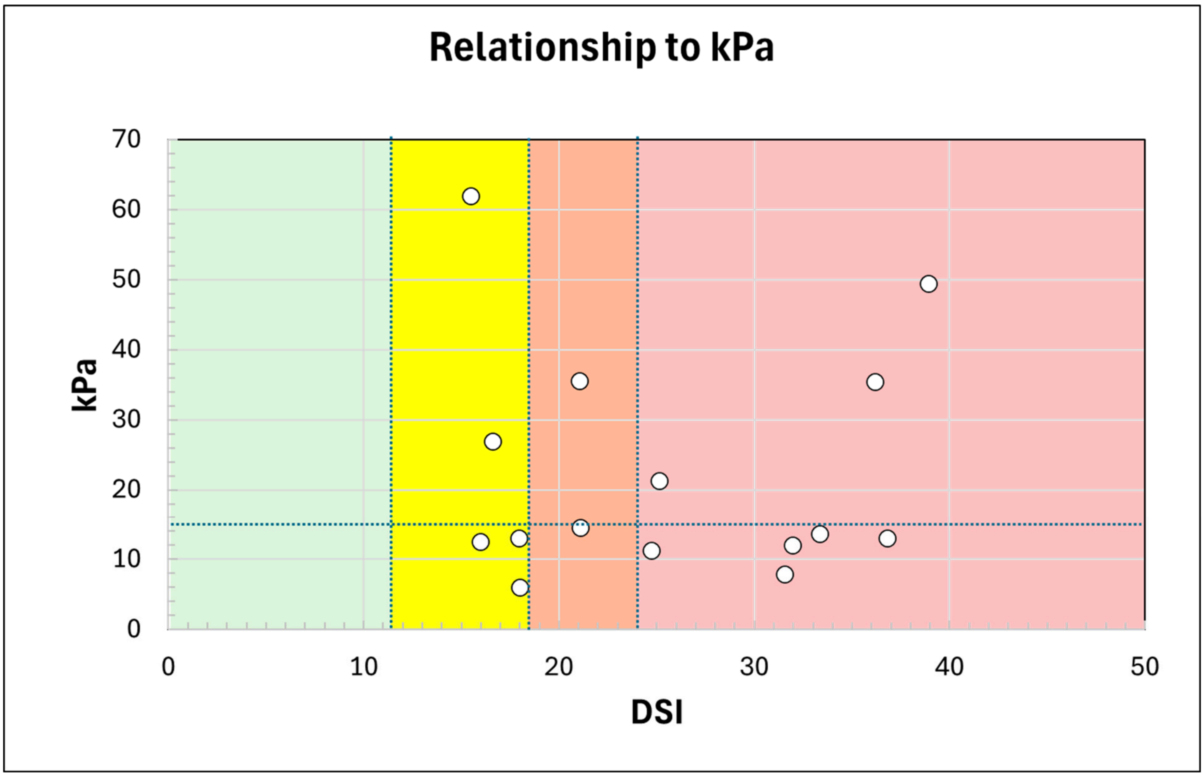

Figure S4. Relationship of DSI to liver stiffness measurement by FibroScan. There was no correlation of kPa from FibroScan with DSI. Of the 5 with high risk (kPa >25) for clinically significant portal hypertension (CSPH) and varices, 2 had either low or intermediate risk DSI. The one with kPa between 15 to 25, indicating high likelihood of cACLD, had intermediate risk DSI. Of the 9 with kPa <15 and low risk for CSPH and avoidance of EGD, 6 had either intermediate or high risk DSI.
